# Supplementary material for: Modern human changes in regulatory regions implicated in cortical development
Source: BMC Genomics. 2020 Apr 16;21:304. doi: 10.1186/s12864-020-6706-x (PMC7161147; doi:10.1186/s12864-020-6706-x)

Supplementary Figures

Figure S1: **Enrichment analyses**. Permutation tests were applied to determine enrichment of enhancers/promoters within human positively-selected regions (n=314, from [8]; overlap = 10 prom; 4 enh), schizophrenia genomic loci (n=108, from [20]; overlap = 2 prom; 3 enh), and autism risk variants (n=58, [21], retrieved from [22]; no overlap).

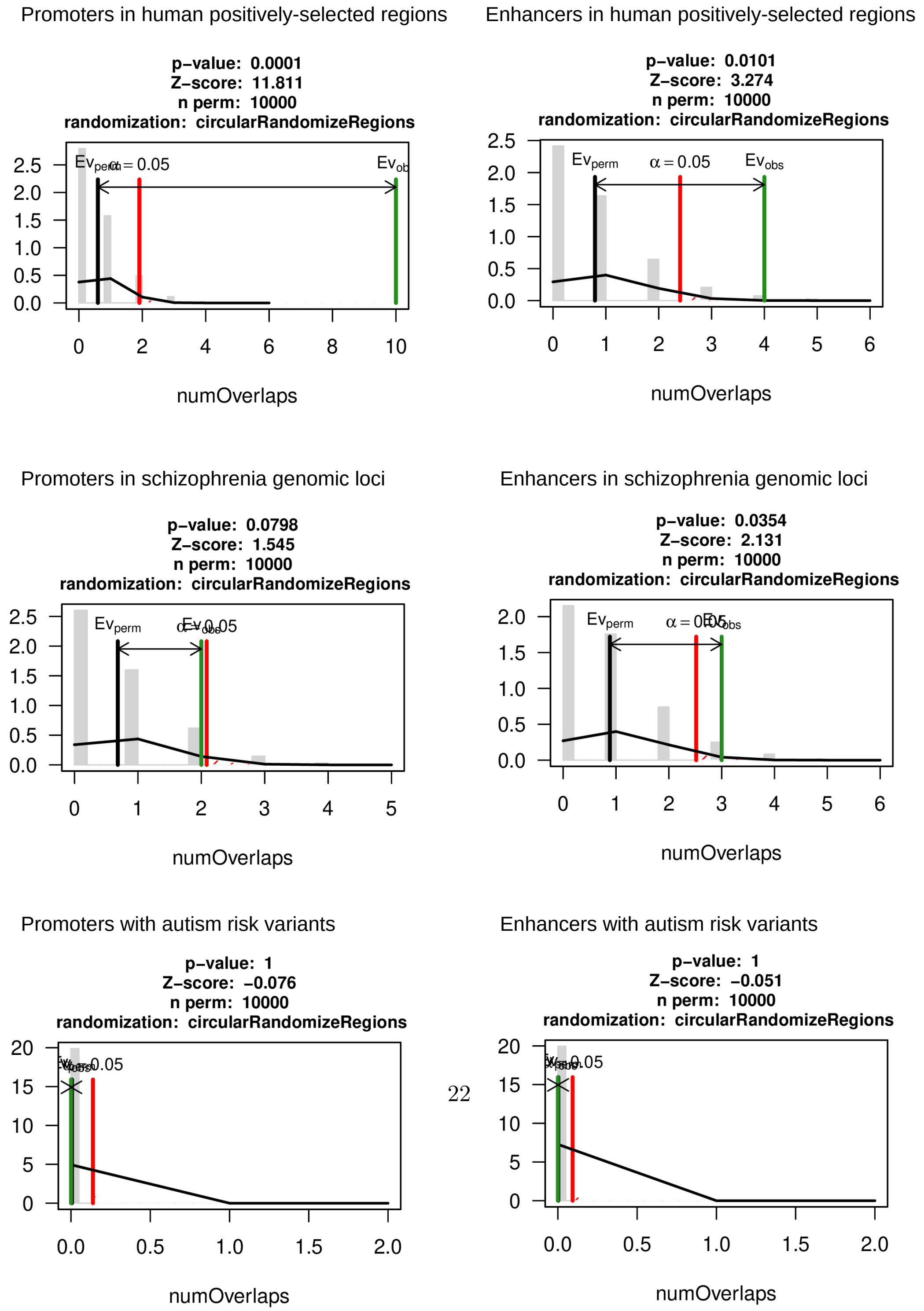

Figure S2: **Clustering results obtained in the original study**. [57] generated and analysed the single-cell transcriptomic data used in this study (5 to 20 post-conception weeks human prenatal brain). They reported two clustering results (A and B) after using different methodologies. Note in B the presence of the three clusters for intermediate progenitor cells and the two clusters of radial glial cells, as in our analysis. Images are reproduced with copyright permission from *Science*.

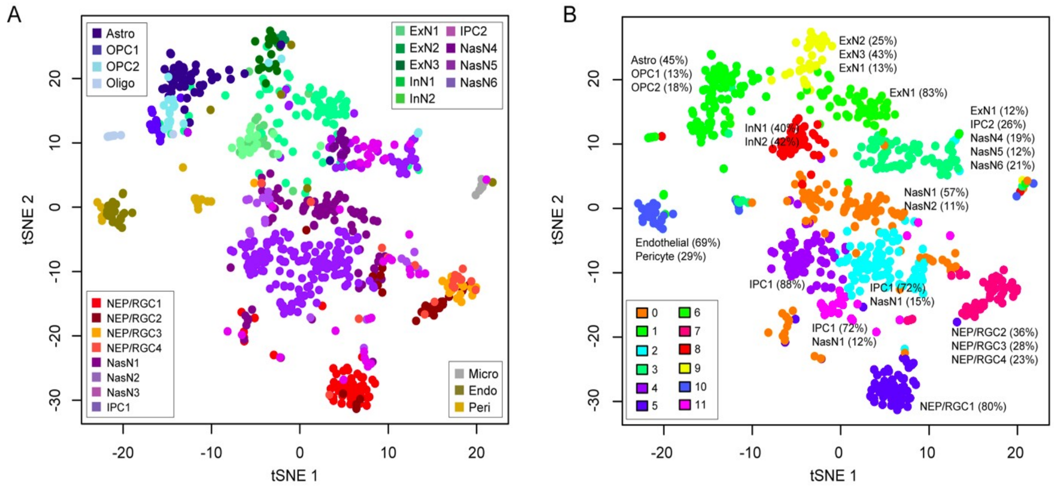

Figure S3: **Co-expression network analysis - Radial glial cells gene modules**. We used the two identified radial glial cell clusters for co-expression analyses. The dendrogram shows genes grouped in different modules (y-axis: distance metrics between clusters) that were assigned a color code (x-axis). A total of 32 modules were detected for RGC-1 sub-population and 26 modules for RGC-cluster 2.

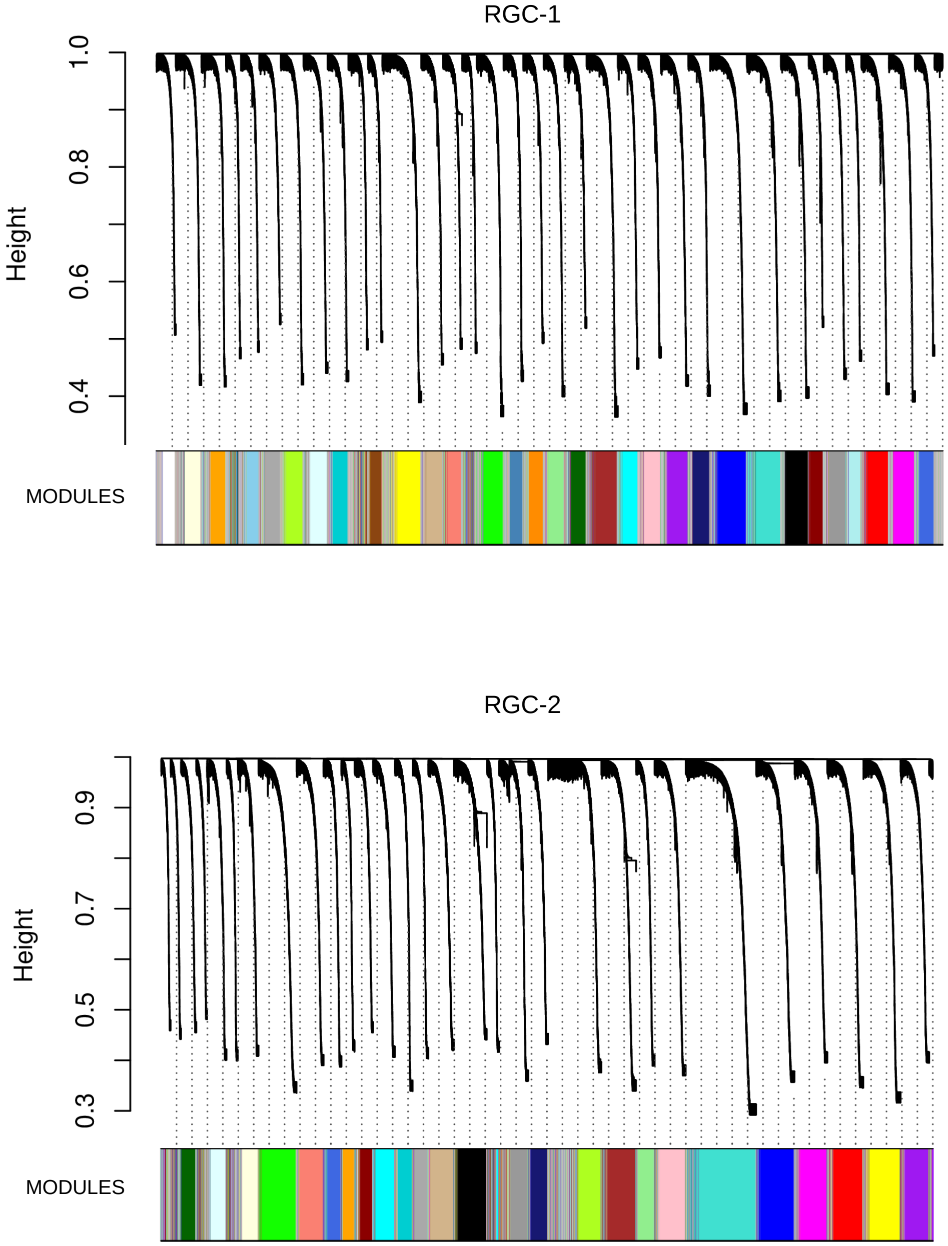

Figure S4: **Co-expression network analysis - Intermediate progenitor cells gene modules**. Two intermediate progenitor cell clusters (IPC-cluster 1 and IPC-cluster 2) were used for co-expression analyses. A total of 9 modules were detected for IPC-cluster 1 and 23 modules for IPC-cluster 2 (y-axis: distance metrics between clusters; x-axis: color code)

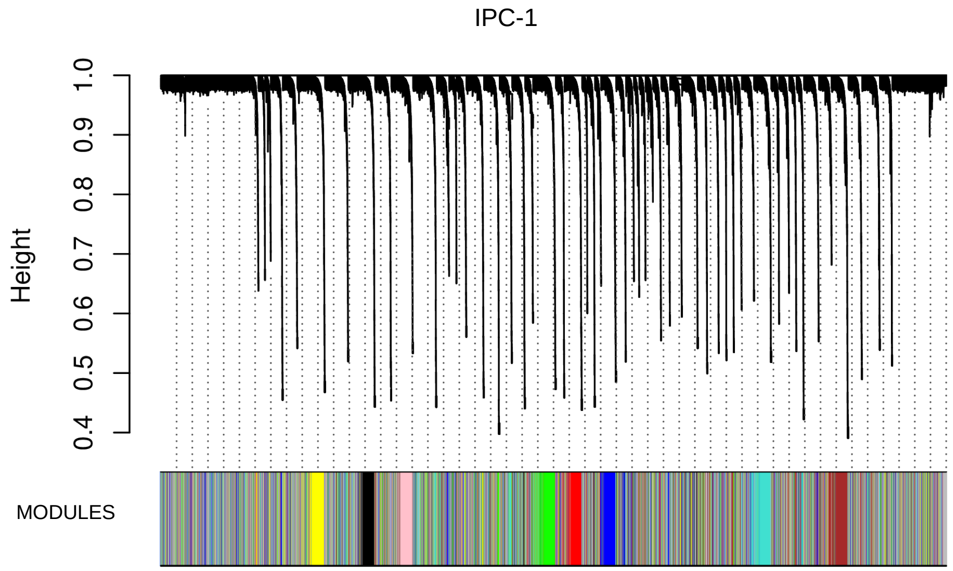

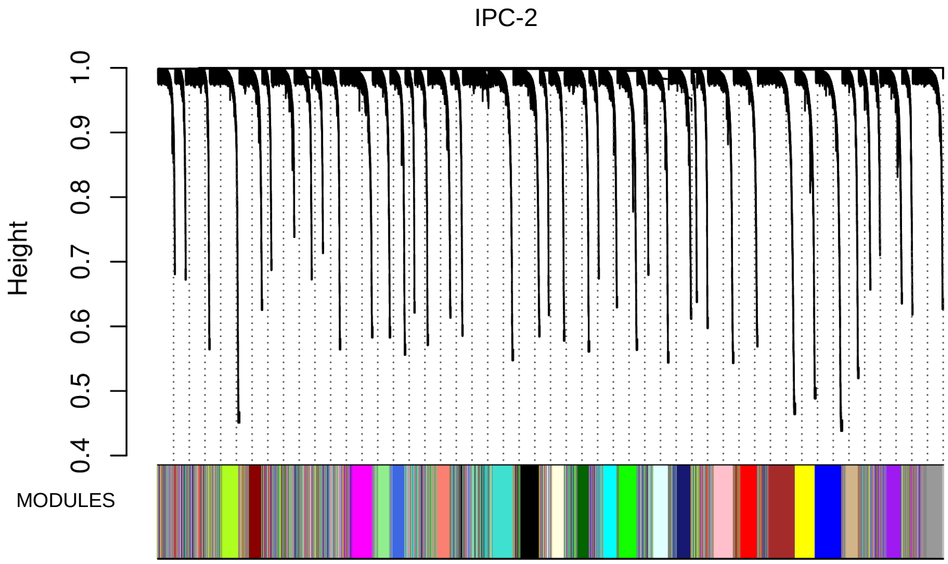

Supplement: Supplementary file 2 — Additional file 2. Supplementary Figures. [file 12864_2020_6706_MOESM2_ESM.pdf]
